# Symptoms of depression and risk of emergency department visits among people aged 70 years and over

**DOI:** 10.1186/s12889-024-17794-6

**Published:** 2024-02-05

**Authors:** Rosamond Dwyer, Kim Jachno, Thach Tran, Alice Owen, Natasha Layton, Taya Collyer, Maggie Kirkman, Judy Lowthian, Karin Hammarberg, John J. McNeil, Robyn L. Woods, Michael Berk, Jane Fisher

**Affiliations:** 1 School of Public Health and Preventive Medicine, Department of Epidemiology and Preventive Medicine, Monash University, Melbourne, VIC Australia; 2 Emergency Department, Peninsula Health, Frankston, VIC Australia; 3 National Centre for Healthy Ageing, Melbourne, Australia; 4 Global and Women’s Health Unit, School of Public Health and Preventive Medicine, Monash University, Melbourne, Australia; 5 Rehabilitation, Ageing and Independent Living (RAIL) Research Centre, Faculty of Medicine, Nursing and Health Sciences, Monash University, Melbourne, VIC Australia; 6 Academic Unit, Peninsula Clinical School, Central Clinical School, Peninsula Health, Monash University, Melbourne, VIC Australia; 7 Bolton Clarke Research Institute, Forrest Hill, VIC Australia; 8 The Institute for Mental and Physical Health and Clinical Translation, School of Medicine, IMPACT, Deakin University, Geelong, Australia; 9 Florey Institute for Neuroscience and Mental Health, University of Melbourne, Melbourne, Australia; 10 Orygen The National Centre of Excellence in Youth Mental Health, Parkville, Australia

**Keywords:** Older persons, Depression, Emergency care, Mental health, Health systems

## Abstract

**Background:**

Older people experiencing depression and anxiety have higher rates of health service utilisation than others, but little is known about whether these influence their seeking of emergency care. The aim was to examine the associations between symptoms of depression and the use of emergency health care, in an Australian context, among a population of people aged 70 years and over initially free of cardiovascular disease, dementia or major physical disability.

**Methods:**

We undertook secondary analyses of data from a large cohort of community-dwelling Australians aged $\ge$70 years. Multivariable logistic regression was used to compare the association of symptoms of depression (measured using the Center for Epidemiological Studies Depression Scale 10 question version, CESD at baseline) with subsequent episodes of emergency care, adjusting for physical and social factors of clinical interest. Marginal adjusted odds ratios were calculated from the logistic regression.

**Results:**

Data were available for 10,837 Australian participants aged at least 70 years. In a follow-up assessment three years after the baseline assessment, 17.6% of people self-reported an episode of emergency care (attended an ED of called an emergency ambulance) in the last 12 months. Use of emergency healthcare was similar for men and women (17.8% vs. 17.4% *p* = 0.61). A score above the cut-off on the CESD at baseline was associated with greater use of emergency health care (OR = 1.35, 95% CI 1.11,1.64). When modelled separately, there was a greater association between a score above the cut-off on the CESD and emergency healthcare for women compared with men.

**Conclusions:**

This study is unique in demonstrating how depressive symptoms among healthy older persons are associated with subsequent increased use of emergency healthcare. Improved understanding and monitoring of mental health in primary care is essential to undertake effective healthcare planning including prevention of needing emergency care.

**Supplementary Information:**

The online version contains supplementary material available at 10.1186/s12889-024-17794-6.

## Introduction

Globally, the population is ageing [1, 2]. Compared with younger populations, older people interact more frequently with the healthcare system [3, 4]. This group of people has complex and specific health needs that are distinct from younger healthcare consumers [5–7].

Presentations to the Emergency Department (ED) of people with recognised and unrecognised mental health concerns are increasing, yet significant gaps remain in understanding the risks for presentation and hospitalisation as well as the recognition, assessment, and management of common mental disorders (CMDs) within the acute care setting [8, 9]. Compared with the broader population, older people experiencing depression and anxiety have been found to have higher rates of health service use across both primary care and tertiary hospital services [10–12]. After an acute hospital admission, they also have higher rates of representation to hospital [10, 12–14]. This heightens their risk of experiencing the adverse effects of inpatient care including iatrogenic injury and functional decline. One study demonstrated that among older people admitted to a medical ward, those with symptoms of depression experienced higher 30-day mortality compared to those without symptoms [12].

Overall, the evidence for care of older people with CMD presenting to the ED is limited. Most studies are from Northern America, where the primary and acute health care systems differ substantially from those in countries with national health services. Each country therefore needs local evidence on which to base policies and practice. Most studies focus on groups with one specific medical diagnosis such as cardiac failure or chronic pulmonary disease rather than considering population-level health concerns and social circumstances. Few studies have examined barriers to detection and management of CMD by healthcare professionals working in a busy and time-pressured emergency department and whether these constitute a risk for high quality care.

The aim of this study was to examine the associations between symptoms of depression and the use of emergency health care, in an Australian context, among a population of people aged 70 years and over initially free of cardiovascular disease, dementia or major physical disability. We hypothesized that older people experiencing symptoms of depression and anxiety were at higher risk of unplanned emergency service use and emergency department attendance and its associated iatrogenic risk.

## Methods

### Design

Secondary analyses of data contributed by a large cohort of community-dwelling healthy Australian participants aged $\ge$70 years at recruitment into the placebo-controlled ASPirin in Reducing Events in the Elderly (ASPREE) clinical trial [15]. Inclusion criteria for ASPREE participants were to be aged at least 70 and willingness to attend a study visit. Exclusion criteria included diagnosis of dementia, independence-limiting physical disability, life limiting illness or prior evidence of cardiovascular disease [15].

### Study population

Approximately 3–6 months after recruitment, ASPREE participants were invited to participate in an associated cohort substudy, the ASPREE Longitudinal Study of Older Persons (ALSOP), by completing a baseline medical questionnaire (*n* = 14,892) (baseline + 3–6 months) which examined general health concerns such as eyesight, hearing, oral health, pain and sleep. An accompanying questionnaire, a baseline social health questionnaire (baseline + 6–9 months), was delivered approximately three months later and captured information on lifestyle and social health such as engagement in recreational and physical activities, social connectedness and use of support services. Follow-up ALSOP Medical and Social questionnaires were completed at years 3 and 5 post enrolment [16].

For inclusion in these secondary analyses, eligible ASPREE participants (*n* = 14,892) needed to have completed all baseline questionnaires for ASPREE and ALSOP (*n* = 12,884) and to have completed the ALSOP Year 3 Social questionnaire (baseline + 36 months) providing the information to assess the main outcome of an episode of emergency care, resulting in a total of 10,837 study participants (Supplementary Figure-1). Of note, ethics stipulated the social questionnaire could not be sent to people who had developed confirmed dementia in the 3-months between initial medical and social questionnaires.

### Outcome ascertainment

The primary outcome was any self-reported attendance at a hospital ED or self-reported ambulance call-out, which we have designated an episode of ‘emergency care’, during the past 12 months ascertained from the Health Services section of the ALSOP Year 3 Social questionnaire.

### Factors associated with emergency healthcare usage

The main exposure factor was the presence of significant depressive symptoms, for which we used scores on the Centre for Epidemiological Studies Depression Scale-10 question version (CES-D-10) collected at ASPREE enrolment (baseline). The CES-D-10 was created as a screening measure for current depressive symptoms. It uses self-report on a four-point ordinal scale (none/rarely, a little/some, occasionally/moderate or most/all of the time) of occurrence during the last week of depressed mood, feelings of guilt, worthlessness or helplessness, loss of appetite, focus or motivation, and sleep difficulties [17]. A cut-off score of > 8 on the CES-D-10 scale was used to designate lower and higher categories of depressive symptoms [18].

### Physical factors

Physical factors at ASPREE baseline examined for associations with an episode of emergency care were age, gender, measures of physical health, polypharmacy, self-reporting of alcohol and smoking status, and from ALSOP, pain or sleep problems, eyesight and hearing problems, having broken a bone or had a fall in the past year. Age was categorized into risk-based quartile groups (70–72 years, 73–75 years, 76–79 years and 80 or more years) based on flexible spline modelling of age against emergency care. Measures of physical health were the Physical Component Summary (PCS) from the Medical Outcomes Study Short Form 12 (SF-12, version 2) [19] and an adapted Fried frailty criteria (not frail, prefrail and frail) which included measures of body weight, hand grip strength, exhaustion, walking speed and physical activity [20, 21]. Polypharmacy was defined as taking more than 5 prescription medications at baseline (yes vs. no), alcohol and smoking status (never, former or current) and the presence of self-reported pain, sleep problems, any history of broken bones and falls in the last year (yes versus no for each condition).

### Social and circumstantial factors

Social and circumstantial factors collected at the time of recruitment into the trial were selected based on results from previous studies [22]. They are, from ASPREE, measures of socioeconomic position, living in a rural or urban area and from ALSOP were housing (living a home they owned, rented or other) and living situation, pet ownership, marital and employment status, support service use, roles as a carer or babysitter, private health insurance, educational attainment, engagement levels with family and friends and in the community more broadly, and self-reporting of major stress in the past year. The postcode for the participant’s residence was used to determine area-level socioeconomic status, the Socio-Economic Indexes for Areas– Index of Relative Socio-Economic Advantage and Disadvantage (SEIFA-IRSAD) [23] and assignment to rural (outer regional or remote) or urban (major cities or inner regional) areas using reference data from the 2011 Australian Census.

### Statistical analysis

Categorical factors were described using frequency (n) and percentages (%) and continuous factors using mean and standard deviation (SD). Univariable chi-square tests for heterogeneity and $t\text{-tests}$ for differences in means were used to assess for difference between participants based on their emergency healthcare usage status for all variables. From the complete list of physical and social factors, the most influential factors for maximizing the distributional balance, or overall comparability of participants likely to be in the higher versus lower depressive groups, were identified by adaptive LASSO (least absolute shrinkage and selection operator) [24, 25]. Gender was identified as an important interaction term for many of the selected factors, so all analyses were stratified and presented separately by gender (female / male). Multivariable logistic regression was then used to compare the association between depressive group membership and episodes of emergency care, adjusting for other physical and social variables. Marginal adjusted odds ratios were reported from the logistic regression.

To further assess the association of depressive symptoms at baseline on risk of experiencing a subsequent episode of emergency care, we used doubly robust treatment effect estimation where the same subset of identified important factors using LASSO selection were used in an inverse probability weighted structural logistic model [26]. After performing the logistic regression, and to allow for valid causal comparisons between the two depressive groups, the risk of using emergency care for each participant was calculated, first assuming the observed depressive group membership, and second using their counterfactual depressive group. The effect of all other observed factors was held constant. The risk in the lower depressive group averaged over all participants was then subtracted from the averaged risk in the higher depressive group yielding the average risk difference. Average risk differences were presented as a percentage difference from the effect observed in the lower depressive group. Because completion of various forms was a requirement for inclusion in this secondary analysis and there was rigorous operational oversight of the data collected during the original clinical trial, there are minimal missing data for any of the outcomes or factors of interest.

### Ethics

Ethics approval for ASPREE and ALSOP was obtained through the Monash University Human Research Ethics Committee (CF07/3730, CF11/1100, CF11/1935). The study was designed and conducted in accordance with the National Health and Medical Research Council Guidelines on Human Experimentation and the Declaration of Helsinki.

## Results

### Demographic, socio-economic and medical characteristics

Data were available for 10,837 Australian participants aged 70-years and over. The mean (SD) age at ASPREE baseline was 75.1(4.2) years, ranging from 70 to 95 years of age for women and 70 to 94 years of age for men. 54.5% of the sample were women. Most participants lived in major cities (54.0%), were English-speaking (96.7%), had completed high school (63.7%), and resided across the spectrum of lower (32.1%), middle (26.4%) and upper (41.5%) socioeconomic areas.

Consistent with requirement for entry to the ASPREE study, this cohort was in good health and living independently. Overall, at ASPREE baseline, 19.4% reported a lifetime history of cancer, 7.4% a lifetime history of diabetes, and 22.5% a lifetime history of depression. 52.8% of participants had an SF12 physical component score above the population standardized average, with a mean (SD) of 48.8(12.2), 24.1% were using five or more prescription medications (defined as polypharmacy). 23% used some home services (including regular home maintenance) and only 1.3% of participants were classified as frail at ASPREE baseline.

### Symptoms of depression and psychological well-being

5.9% of people scored above the cut-off of 8 for symptoms of depression using the CESD − 10, with a median score of 2 (IQR 1–4). Men reported fewer depressive symptoms than women (median 2, IQR 0–4, vs. median 2, IQR 1–5, *p* < 0.001). From the initial ALSOP questionnaire data, overall, 99.6% of people described good engagement with family and friends and 80.4% were categorized as having high levels of community engagement. Only 13.5% reported a significant carer burden. At baseline 53.3% reported a major stressful life event over the preceding 12-months.

On entry to the study, 17.5% of participants reported significant pain, with women (21.0%) being more likely to report pain than men (13.4%; *p* < 0.001). More than one third of participants (36%) described frequent sleep disturbance, which was more common in women (39.5%) than men (31.8%; *p* < 0.001).

### Emergency service use

In all, 17.6% of people self-reported experiencing an episode of emergency care (attended an ED or called an emergency ambulance) in the 12 months prior to completing the Year Three ALSOP questionnaire. Use of emergency healthcare was similar for men and women (17.8% vs. 17.4% *p* = 0.61). The proportion of people using emergency health services was greater for those in higher age brackets, increasing from 15.1% for those aged 70–72 years to 22.8% for participants aged 80 + at baseline; *p* < 0.001). Tables 1 and 2 show ASPREE baseline characteristics including data from the initial ALSOP questionnaires for men and women in the ASPREE/ALSOP cohort with bivariate assessment of association with emergency care. Results are presented separately for women and men. The baseline characteristics of all participants are presented in Supplementary Table 1.

**Table 1:** Baseline/initial physical characteristics and symptoms of depression for participants in the ASPREE/ALSOP cohort with univariate assessment of association with emergency care usage

				Males (*N* = 4933)				Females (*N* = 5904)		
				Emergency service use		***p***-value		Emergency service use		***p***-value
				No	Yes			No	Yes	
***Depressive symptoms indication***										
	CESD ≤ 8			3869 (95.4%)	815 (93.0%)	0.004		4586 (94.0%)	922 (89.9%)	< 0.001
	CESD > 8			188 (4.6%)	61 (7.0%)			289 (5.9%)	104 (10.1%)	
***Biological factors***										
	Age group, n (%)									
		70–72 years		1774 (43.7%)	318 (36.3%)	< 0.001		1988 (40.8%)	350 (34.1%)	< 0.001
		73–75 years		1059 (26.1%)	215 (24.5%)			1289 (26.4%)	277 (27.0%)	
		76–79 years		693 (17.1%)	186 (21.2%)			947 (19.4%)	207 (20.2%)	
		80 + years		531 (13.1%)	157 (17.9%)			654 (13.4%)	192 (18.7%)	
	Smoking status, n (%)									
		Current		115 (2.8%)	34 (3.9%)	0.13		107 (2.2%)	17 (1.7%)	0.01
		Former		2150 (53.0%)	478 (54.6%)			1461 (30.0%)	353 (34.4%)	
		Never		1792 (44.2%)	364 (41.6%)			3310 (67.9%)	656 (63.9%)	
	Alcohol use, n (%)									
		Current		3509 (86.5%)	754 (86.1%)	0.78		3674 (75.3%)	764 (74.5%)	0.27
		Former		205 (5.1%)	42 (4.8%)			172 (3.5%)	47 (4.6%)	
		Never		343 (8.5%)	80 (9.1%)			1032 (21.2%)	215 (21.0%)	
	Frailty, n (%)									
		Not frail		2698 (66.5%)	510 (58.2%)	< 0.001		3185 (65.3%)	601 (58.6%)	< 0.001
		Pre-frail		1312 (32.3%)	355 (40.5%)			1633 (33.5%)	405 (39.5%)	
		Frail		47 (1.2%)	11 (1.3%)			60 (1.2%)	20 (1.9%)	
	Physical component score, mean (sd)			50.1 (7.6)	48.0 (8.5)	< 0.001		48.4 (8.7)	46.3 (9.4)	< 0.001
	Polypharmacy, n (%)									
		No		3365 (82.9%)	665 (75.9%)	< 0.001		3561 (73.0%)	634 (61.8%)	< 0.001
		Yes		692 (17.1%)	211 (24.1%)			1317 (27.0%)	392 (38.2%)	
	Significant sleep problems*, n (%)									
		No		2773 (68.4%)	563 (64.3%)	0.05		2962 (60.7%)	584 (56.9%)	0.05
		Yes		1251 (30.8%)	303 (34.6%)			1881 (38.6%)	431 (42.0%)	
	Significant pain problems*, n (%)									
		No		3548 (87.5%)	723 (82.5%)	< 0.001		3927 (80.5%)	739 (72.0%)	< 0.001
		Yes		509 (12.5%)	153 (17.5%)			951 (19.5%)	287 (28.0%)	
	Good eyesight*, n (%)									
		No		740 (18.2%)	176 (20.1%)	0.2		885 (18.1%)	222 (21.6%)	0.02
		Yes		3251 (80.1%)	691 (78.9%)			3921 (80.4%)	786 (76.6%)	
	Hearing problems*, n (%)									
		No		1729 (42.6%)	335 (38.2%)	0.05		2885 (59.1%)	565 (55.1%)	0.05
		Yes		2174 (53.6%)	508 (58.0%)			1765 (36.2%)	405 (39.5%)	
	Ever broken bone(s)*, n (%)									
		No		2480 (61.1%)	497 (56.7%)	0.01		2997 (61.4%)	575 (56.0%)	0.005
		Yes		1485 (36.6%)	348 (39.7%)			1771 (36.3%)	423 (41.2%)	
	Falls in past year*, n (%)									
		No		3114 (76.8%)	613 (70.0%)	< 0.001		3301 (67.7%)	633 (61.7%)	< 0.001
		Yes		919 (22.7%)	255 (29.1%)			1542 (31.6%)	379 (36.9%)	

*from initial ALSOP questions, remainder of variables were from ASPREE at baseline

**Table 2:** Baseline/initial social characteristics for participants in the ASPREE/ALSOP cohort with univariate assessment of association with emergency care usage

				Males (*N* = 4933)				Females (*N* = 5904)		
				Emergency service use		***p***-value		Emergency service use		***p***-value
				No	Yes			No	Yes	
***Social factors***										
	Socio-economic position, n (%)									
		Lower (deciles 1–4)		1252 (30.9%)	314 (35.8%)	0.001		1538 (31.5%)	372 (36.3%)	0.03
		Middle (deciles 5–7)		1045 (25.8%)	236 (26.9%)			1321 (27.1%)	249 (24.3%)	
		Upper (deciles 8–10)		1755 (43.3%)	322 (36.8%)			2007 (41.1%)	403 (39.3%)	
	Rurality, n (%)									
		Outside a major city		1831 (45.1%)	440 (50.2%)	0.002		2189 (44.9%)	517 (50.4%)	0.005
		Major city		2221 (54.7%)	432 (49.3%)			2677 (54.9%)	507 (49.4%)	
	Accommodation type*, n (%)									
		Not house		75 (1.8%)	27 (3.1%)	0.07		88 (1.8%)	34 (3.3%)	0.008
		House		3927 (96.8%)	838 (95.7%)			4686 (96.1%)	972 (94.7%)	
	Home ownership*, n (%)									
		Not owned by participant		279 (6.9%)	88 (10.0%)	0.005		463 (9.5%)	113 (11.0%)	0.23
		Owned by participant		3718 (91.6%)	775 (88.5%)			4302 (88.2%)	894 (87.1%)	
	Living arrangement*, n (%)									
		Lives with others		3405 (83.9%)	707 (80.7%)	0.06		2899 (59.4%)	542 (52.8%)	< 0.001
		Lives alone		620 (15.3%)	162 (18.5%)			1932 (39.6%)	475 (46.3%)	
	Has a pet*, n (%)									
		No		2523 (62.2%)	512 (58.4%)	0.11		3086 (63.3%)	674 (65.7%)	0.34
		Yes		1457 (35.9%)	347 (39.6%)			1718 (35.2%)	337 (32.8%)	
	In paid work*, n (%)									
		No		3431 (84.6%)	755 (86.2%)	0.20		4466 (91.6%)	931 (90.7%)	0.53
		Yes		533 (13.1%)	97 (11.1%)			276 (5.7%)	60 (5.8%)	
	Has carer burden*, n (%)									
		No		3546 (87.4%)	748 (85.4%)	0.24		3987 (81.7%)	796 (77.6%)	< 0.001
		Yes		421 (10.4%)	103 (11.8%)			726 (14.9%)	167 (16.3%)	
	Provides regular babysitting*, n (%)									
		No		3632 (89.5%)	804 (91.8%)	0.11		4143 (84.9%)	862 (84.0%)	0.001
		Yes		344 (8.5%)	56 (6.4%)			615 (12.6%)	117 (11.4%)	
	Support service usage*, n (%)									
		No		3462 (85.3%)	676 (77.2%)	< 0.001		3518 (72.1%)	655 (63.8%)	< 0.001
		Yes		588 (14.5%)	198 (22.6%)			1342 (27.5%)	371 (36.2%)	
	Private health insurance*, n (%)									
		No		1149 (28.3%)	332 (37.9%)	< 0.001		1526 (31.3%)	374 (36.5%)	0.001
		Yes		2908 (71.7%)	544 (62.1%)			3352 (68.7%)	652 (63.5%)	
	Married*, n (%)									
		No		752 (18.5%)	193 (22.0%)	0.02		2277 (46.7%)	532 (51.9%)	0.003
		Yes		3305 (81.5%)	683 (78.0%)			2601 (53.3%)	494 (48.1%)	
	Completed high school*, n (%)									
		No		1273 (31.4%)	295 (33.7%)	0.42		1882 (38.6%)	432 (42.1%)	0.04
		Yes		2727 (67.2%)	569 (65.0%)			2941 (60.3%)	578 (56.3%)	
	Community engagement levels*, n (%)									
		Low		967 (23.8%)	211 (24.1%)	0.94		744 (15.3%)	157 (15.3%)	0.03
		High		3014 (74.3%)	650 (74.2%)			4030 (82.6%)	833 (81.2%)	
	Family & friends engagement*, n (%)									
		Low		23 (0.6%)	8 (0.9%)	0.49		10 (0.2%)	4 (0.04%)	0.08
		High		4026 (99.2%)	866 (98.9%)			4850 (99.4%)	1022 (99.6%)	
	Had major stress in past year*, n (%)									
		No		2050 (50.5%)	394 (45.0%)	0.003		2176 (44.6%)	445 (43.4%)	0.47
		Yes		2007 (49.5%)	482 (55.0%)			2702 (55.4%)	581 (56.6%)	

*from initial ALSOP questions, remainder of variables were from ASPREE at baseline

### Multivariate logistic and causal modelling

The results of the multivariable logistic regression analysis of emergency care usage for the covariates stratified by sex are presented in Table 3. When modelled separately, there was a significantly greater association between a score above the cut-off on the CESD and emergency healthcare use for women compared with men (OR 1.45, 95% CI 1.13–1.87 vs. OR 1.22, 95% CI 0.89–1.68). The positive association between the poorer physical function score and recent history of falls with emergency healthcare use remained statistically significant for men, whereas polypharmacy and pain problems remained significant for women. Living in a major city, living with others, and residing in their own home were associated with less emergency healthcare use for women but not men.

**Table 3:** Multivariable logistic regression analysis of emergency care usage using factors selected by adaptive LASSO, stratified by sex

				Males (*N* = 4741)				Females (*N* = 5638)		
				OR	95% CI	***p***-value		OR	95% CI	***p***-value
***Depressive symptoms indication***										
	CESD ≤ 8			Reference				Reference		
	CESD > 8			1.22	0.89, 1.68	0.22		1.45	1.13, 1.87	0.004
***Biological factors***										
	Age group									
		70–72 years		Reference				Reference		
		73–75 years		1.07	0.88, 1.30	0.51		1.15	0.96, 1.38	0.12
		76–79 years		1.37	1.11, 1.69	0.003		1.08	0.88, 1.32	0.45
		80 + years		1.34	1.06, 1.70	0.01		1.37	1.10, 1.70	0.005
	Physical component score			0.88	0.81, 0.96	0.005		0.94	0.88, 1.02	0.14
	Polypharmacy									
		No		Reference				Reference		
		Yes		1.26	1.04, 1.52	0.02		1.40	1.19, 1.63	< 0.001
	Significant pain problems									
		No		Reference				Reference		
		Yes		1.08	0.86, 1.35	0.50		1.25	1.05, 1.51	0.01
	Falls in past year									
		No		Reference				Reference		
		Yes		1.23	1.04, 1.46	0.02		1.15	0.99, 1.33	0.06
***Social factors***										
	Socio-economic status									
		Lower (deciles 1–4)		Reference				Reference		
		Middle (deciles 5–7)		0.95	0.78, 1.16	0.64		0.82	0.68, 0.99	0.04
		Upper (deciles 8–10)		0.90	0.72, 1.11	0.32		1.03	0.85, 1.26	0.74
0.74222222										
	Rurality, n (%)									
		Outside a major city		Reference				Reference		
		Major city		0.92	0.77, 1.11	0.39		0.78	0.65, 0.92	0.003
	Accommodation type									
		Not house		Reference				Reference		
		House		0.65	0.41, 1.03	0.06		0.58	0.39, 0.88	0.01
	Living arrangement									
		Lives with others		Reference				Reference		
		Lives alone		1.03	0.84, 1.27	0.79		1.16	1.00, 1.34	0.05
	Has a pet									
		No		Reference				Reference		
		Yes		1.11	0.94, 1.30	0.21		0.88	0.75, 1.02	0.09
	Support service usage									
		No		Reference				Reference		
		Yes		1.41	1.15, 1.72	0.001		1.18	1.00, 1.38	0.05
	Private health insurance									
		No		Reference				Reference		
		Yes		0.73	0.62, 0.86	< 0.001		0.85	0.73, 0.99	0.03
	Family & friends engagement									
		Low		Reference				Reference		
		High		0.67	0.29, 1.54	0.34		0.70	0.22, 2.27	0.55

When all participants were modelled regardless of gender, a score above the cut-off on the CESD was associated with greater use of emergency health care (OR = 1.35, 95% CI 1.11,1.64; Supplementary Table 2). Other variables associated with increased likelihood of emergency healthcare use were polypharmacy (OR = 1.33, 95% CI 1.04,1.52), use of support services (OR 1.27, 95% CI 1.12–1.44) and having had a fall within the last 12 months (OR = 1.18 95% CI 1.03–1.32). Emergency healthcare use was less likely for those with better physical function (OR = 0.88, 95% CI 0.81 1.30), living in a major city (OR 0.84, 95% CI 0.74–0.95), with private health insurance (OR 0.79, 95% CI 0.71–0.89) and living in their own home (OR 0.6 95% CI 0.44–0.82).

To quantify the association between depressive symptoms and the use of emergency care, the risk differences were estimated using doubly robust structural models appropriate for causal inference (Table 4). Overall, the proportion of participants with no or low depressive symptoms at baseline who used emergency care was 17.1% (95% CI 16.4%, 17.8%). The average risk difference in all participants with depressive symptoms at baseline was an estimated increase of 5.2% (95% CI 1.6%, 8.7%; *p* = 0.004) in the proportion of participants experiencing an episode of emergency care.

**Table 4:** Causal inference analysis of proportions of older adults reporting an episode of emergency care by depressive symptom group. Selection of optimal factors (and all possible interactions) to obtain overall balance between depressive group assignment was restricted to the factors identified used in Table 2

	**Overall**	Stratified by sex	
		Males	Females
No or low depressive symptoms CESD ≤ 8	17.1% (16.4%, 17.8%)	17.1% (16.2%, 18.4%)	16.8% (15.8%, 17.8%)
Higher depressive symptoms CESD > 8	22.3% (18.8%, 25.8%)	24.5% (19.0%, 30.0%)	20.7% (16.4%, 25.1%)
Mean difference	5.2% (1.6%, 8.7%) *p* = 0.004	7.1% (1.6%, 12.7%) *p* = 0.01	3.9% (-0.5%, 8.3%) *p* = 0.08

When modelled separately by sex, male participants in the no or low depressive symptoms group had a 17.1% (16.2%, 18.4%) chance of utilizing emergency care while those in the higher depressive symptoms group had an estimated increased usage of 7.1% (1.6%, 12.7%; *p* = 0.01). The estimated association of the depressive symptoms group was not as large in women, with participants in the no or low group having a mean 16.8% (15.8%, 17.8%) proportion at baseline compared to 20.7% (16.4%, 25.1%) participants in the high group. This results in an estimated increased proportion of 3.9% (-0.5%, 8.3%; *p* = 0.08) for women that could be attributed to being in the higher depressive group.

## Discussion

To our knowledge, this is the first study to describe the relationship between symptoms of depression and subsequent emergency healthcare use in a population of healthy Australians aged at least 70 years. Our results demonstrate a strong statistically and clinically significant positive association between symptoms of depression and later use of emergency healthcare services. These findings are consistent with previous studies demonstrating more frequent emergency department attendances, reattendances and unplanned hospitalizations for people with symptoms of depression among sub-groups of the population living with chronic disease [14, 27, 28]. Given the origins of our study population, in a clinical trial which excluded those with with major morbidities of dementia, physical disability or cardiovascular disease, this group of people may be considered healthier and more independent than the general population of older adults [22]. It is therefore possible that our findings are an underestimation of the true prevalence of symptoms of depression and the association between these symptoms and emergency healthcare use among the broader population.

Our findings are consistent with existing evidence that some physical features are associated with increased risk of requiring emergency care [7]. These include physical frailty, recurrent falls, and polypharmacy. Among the socioeconomic variables we did not find correlations between some factors often thought to be protective such as living with other people, relationships with family and friends, and community engagement. In this study this could reflect an insensitive method of data collection that does not capture the true nature of these features. Additionally, our study cohort members were relatively uniform in their independence, socioeconomic position and social engagement which may have prevented an association being found between these variables and healthcare use. A recent review by Valtorta et al. [29], in which the relationship between social relationships, social isolation and health service use was examined, was unable to establish a consistent association between weaker social relationships and emergency department attendances in the general population. The authors did find that the association between weaker social relationships and increased health service use became stronger for people who were already experiencing illness by being associated with poorer medication compliance and decreased coping mechanisms [29]. This may explain why we did not find these associations in our population of relatively well people.

Studies in emergency departments and inpatient hospital environments have described the considerable burden of symptoms of depression and anxiety in patients that are not detected by healthcare professions [9, 12]. This may relate to cognitive bias of healthcare workers and fixation on physical symptoms, time-pressured environments, or lack of specific education about these conditions [9]. In addition, a high proportion of older people presenting to hospital with depression and loneliness have been found, to present predominantly with non-specific somatic symptoms of emotional distress such as chest pain, fatigue, back pain and dizziness [11, 30, 31]. These factors may complicate the initial assessment and obscure the underlying mental health diagnosis [9]. A routinely conducted screening tool may be an easy, objective and potentially time efficient way of detecting these concerns and may be conducted in a preventative approach in primary healthcare or to assist in comprehensive care in an emergency setting. It would be important to examine the impact of such an intervention on subsequent risk of unplanned hospital presentation.

Among older people, correlates of depression include diverse social, psychological, biological and healthcare factors. There may be varied clinical expression of symptoms and detection and management can be complex [32]. Together our findings indicate that screening people aged 70-years and over for early detection of symptoms of depression and anxiety in primary care may allow for interventions to mitigate the risk of requiring emergency care in the future. Ideally this would commence as part of accessible, comprehensive primary healthcare. Findings also reinforce the need for healthcare professionals to consistently re-evaluate a person’s social and economic circumstances in the setting of acute or evolving illness, where these may become more important in provision of holistic, effective healthcare and ideally as part of accessible, comprehensive primary care.

Results of this study include important observed differences between women and men. This is consistent with previous studies describing differing experience of both physical and psychological ill-health according to gender [33–35]. In our study a score above the cut-off on the CESD, living alone, living outside major city and chronic pain were all more closely associated with emergency healthcare use for women than men. These findings suggest different social circumstances and physical health risk factors between genders as well as variations in depressive symptoms. Improving access to primary healthcare, especially in rural settings and addressing gender differences in healthcare seeking behaviour may positively influence the detection and management of psychological mental health symptoms. This reinforces the strong need for gender informed screening processes and person-orientated approaches to healthcare and care planning.

### Strengths and limitations

As a cohort study we cannot draw absolute conclusions of causality since estimation of effects from observational data may be subject to biases from confounding, selection and measurement error when conducting statistical modelling. It is also important to note the CESD is a measure of symptoms at a single moment in time (3 years prior to assessment of the outcome) and not necessarily reflective of ongoing experience of symptoms in the longer term.

In order to minimize these potential biases, we undertook a design-based approach to analysis, applying a framework for the thoughtful application of multiple approaches as advocated when undertaking observational research with causal goals [36]. Our findings, using this framework, allowed for an agnostic selection of influential covariates and aligned with results obtained using more conventional statistical approaches. Additionally, we had a very large selection of available baseline potential confounders and the CESD-10 assessment of symptoms of depression was completed three years prior to when emergency healthcare use was ascertained. The temporal structure available in these data further strengthens the evidence that having symptoms of depression in older people contributes significantly to the risk of future use of emergency care. ASPREE treatment allocation was not included as a separate covariate, it is worth noting the overall findings of ASPREE were that regular low dose aspirin did not improve disability-free survival [15].

A key strength of this study was access to the data from a large cohort of older persons in Australia with comprehensive data collection and high retention across assessment waves. In addition, this study sample included people living in both major cities and more rural settings thus these findings represent people living across diverse locations. Eligibility for the ASPREE/ALSOP cohort was restricted to relatively healthy participants by design, so findings may not be fully representative of the broader population of older persons in Australia, and of older persons in other countries. The comprehensive data available enabled us to undertake an analysis within a causal inference framework that aims to address the potential biases that can arise in observational data. This allowed us to obtain estimates of effects of depressive symptoms for men and women of similar direction and magnitude as that seen in the more classical modelling approaches.

Overall, the participants reported baseline levels of depressive symptoms that were at the lower end of the CESD-10 depressive symptom scale, again likely to be the result of the design of the RCT which provided these cohort data. The threshold used to ascertain the self-reported depressive symptoms group assignment was based on previous use of the scale in this cohort. However, the inherently imperfect nature of self-report and the relative good health of these volunteers meant that our findings were not robust to changes in the threshold cut-off points. Sensitivity analyses of alternative cut off points resulted in less consistent associations which were driven by the lower power of either increased homogeneity or smaller group size. Longer follow up of this relatively healthy cohort would provide the opportunity to assess the contribution of longitudinal changes in depressive symptoms on the use of emergency care and is being undertaken. Our outcome of emergency care is broad and we were not able to differentiate the cause or outcome of these presentations. Data linkage of ambulance usage with hospital admissions is planned which will enable a more robust non-subjective assessment of the measurement of the emergency aspects of health care utilization. In addition, it will be important to examine, using prospective and qualitative methods the impact of an emergency department visit on a person’s psychological state during an admission and after leaving hospital, where a sense of vulnerability and apprehension may heighten symptoms of depression and anxiety.

## Conclusion

Frequent episodes of emergency care may have adverse consequences for both individuals and the healthcare system. This study is unique in demonstrating how depressive symptoms among healthy older persons are associated with increased subsequent of emergency healthcare. Improved understanding and monitoring of social and emotional wellbeing are essential to undertake effective healthcare planning and in establishing safety nets in anticipation of acute illness. This requires effective collaboration and communication between clinicians located in the community, primary care, and hospitals. Further studies are required to examine the most successful methods of health information transmission between the primary care, older consumer and emergency settings and how best to translate this to improved psychologically informed healthcare for older people.

### Electronic supplementary material

Below is the link to the electronic supplementary material.

Supplementary Material 1

## Data Availability

The data that support the findings of this study are available from the ASPREE Project but restrictions apply to the availability of these data, which were used under license for the current study, and so are not publicly available. Data are however available from the authors upon reasonable request and with permission of the ASPREE Project Committee, contact person Dr Alice Own, alice.owen@monash.edu.
