# Supplementary material for: Symptoms of depression and risk of emergency department visits among people aged 70 years and over
Source: BMC Public Health. 2024 Feb 5;24:385. doi: 10.1186/s12889-024-17794-6 (PMC10845391; doi:10.1186/s12889-024-17794-6)
Supplement: Supplementary file 1 — Supplementary Material 1 [file 12889_2024_17794_MOESM1_ESM.docx]

**SUPPLEMENTARY MATERIAL**

Supplementary Figure 1: Inclusion into subanalysis

**
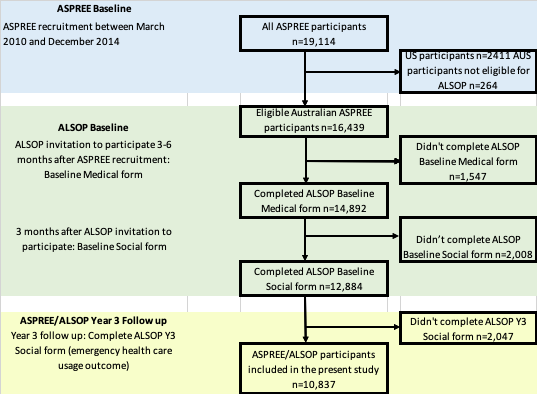
**

| **Supplementary Table 1:** Baseline characteristics in the ASPREE/ALSOP cohort with univariate assessment of association with emergency care usage | | | | | |
| --- | --- | --- | --- | --- | --- |
|  |  |  |  |  |  |
|  |  |  | ASPREE/ALSOP cohort N=10837 | | |
|  |  |  | Didn't use emergency care | Used emergency care | χ^2^-square or t-test p-value |
|  |  |  | Number (%) / Mean ± SD | Number (%) / Mean ± SD |  |
|  |  |  |  |  |  |
| Depressive symptoms | | |  |  |  |
|  | CESD≤8 | | 8455 (94.6%) | 1737 (91.3%) | <0.001 |
|  | CESD>8 | | 477 (5.3%) | 165 (8.7%) |  |
|  | Missing | | 3 (0.03%) | 0 (0.0%) |  |
|  |  |  |  |  |  |
| Biological factors | | |  |  |  |
|  | Age group | |  |  |  |
|  |  | 70-72 | 3762 (42.1%) | 668 (35.1%) | <0.001 |
|  |  | 73-75 | 2348 (26.3%) | 492 (25.9%) |  |
|  |  | 76-79 | 1640 (18.4%) | 393 (20.7%) |  |
|  |  | 80+ | 1185 (13.3%) | 349 (18.3%) |  |
|  |  |  |  |  |  |
|  | Gender | |  |  |  |
|  |  | Male | 4057 (45.4%) | 876 (46.1%) | 0.60 |
|  |  | Female | 4878 (54.6%) | 1026 (53.9%) |  |
|  |  |  |  |  |  |
|  | Smoking status | |  |  |  |
|  |  | Current | 222 (2.5%) | 51 (2.7%) | 0.02 |
|  |  | Former | 3611 (40.4%) | 831 (43.7%) |  |
|  |  | Never | 5102 (57.1%) | 1020 (53.6%) |  |
|  |  |  |  |  |  |
|  | Alcohol use | |  |  |  |
|  |  | Current | 7183 (80.4%) | 1518 (79.8%) | 0.65 |
|  |  | Former | 377 (4.2%) | 89 (4.7%) |  |
|  |  | Never | 1375 (15.4%) | 295 (15.5%) |  |
|  |  |  |  |  |  |
|  | Frailty | |  |  |  |
|  |  | Not | 5883 (65.8%) | 1111 (58.4%) | <0.001 |
|  |  | Prefrail | 2945 (33.0%) | 760 (40.0%) |  |
|  |  | Frail | 107 (1.2%) | 31 (1.6%) |  |
|  |  |  |  |  |  |
|  | PCS |  | 49.1 ± 8.3 | 47.1 ± 9.1 | <0.001 |
|  |  | Missing | 3 | 2 |  |
|  |  |  |  |  |  |
|  | Polypharmacy >5 | |  |  |  |
|  |  | No | 6926 (77.5%) | 1299 (68.3%) | <0.001 |
|  |  | Yes | 2009 (22.5%) | 603 (31.7%) |  |
|  |  |  |  |  |  |
|  | Significant sleep problems | |  |  |  |
|  |  | No | 5735 (64.2%) | 1147 (60.3%) | 0.003 |
|  |  | Yes | 3132 (35.1%) | 734 (38.6%) |  |
|  |  | Missing | 68 (0.8%) | 21 (1.1%) |  |
|  |  |  |  |  |  |
|  | Significant pain problems | |  |  |  |
|  |  | No | 7475 (83.7%) | 1462 (76.9%) | <0.001 |
|  |  | Yes | 1460 (16.3%) | 440 (23.1%) |  |
|  |  |  |  |  |  |
|  | Good eyesight | |  |  |  |
|  |  | No | 1625 (18.2%) | 398 (20.9%) | 0.02 |
|  |  | Yes | 7172 (80.3%) | 1477 (77.7%) |  |
|  |  | Missing | 138 (1.5%) | 27 (1.4%) |  |
|  |  |  |  |  |  |
|  | Hearing problems | |  |  |  |
|  |  | No | 4614 (51.6%) | 900 (47.3%) | 0.003 |
|  |  | Yes | 3939 (44.1%) | 913 (48.0%) |  |
|  |  | Missing | 382 (4.3%) | 89 (4.7%) |  |
|  |  |  |  |  |  |
|  | Ever broken bone(s) | |  |  |  |
|  |  | No | 5477 (61.3%) | 1072 (56.4%) | <0.001 |
|  |  | Yes | 3256 (36.4%) | 771 (40.5%) |  |
|  |  | Missing | 202 (2.3%) | 59 (3.1%) |  |
|  |  |  |  |  |  |
|  | Falls in past year | |  |  |  |
|  |  | No | 6415 (71.8%) | 1246 (65.5%) | <0.001 |
|  |  | Yes | 2461 (27.5%) | 634 (33.3%) |  |
|  |  | Missing | 59 (0.7%) | 22 (1.2%) |  |
|  |  |  |  |  |  |
| Social factors | |  |  |  |  |
|  | SES |  |  |  |  |
|  |  | Lower 1-4 | 2790 (31.2%) | 686 (36.1%) | <0.001 |
|  |  | Middle 5-7 | 2366 (26.5%) | 485 (25.5%) |  |
|  |  | Upper 8-10 | 3762 (42.1%) | 725 (38.1%) |  |
|  |  | Missing | 17 (0.2%) | 6 (0.3%) |  |
|  |  |  |  |  |  |
|  | Rurality | |  |  |  |
|  |  | Not major city | 4020 (45.0%) | 957 (50.3%) | <0.001 |
|  |  | Major city | 4898 (54.8%) | 939 (49.4%) |  |
|  |  | Missing | 17 (0.2%) | 6 (0.3%) |  |
|  |  |  |  |  |  |
|  | House type | |  |  |  |
|  |  | Not house | 163 (1.8%) | 61 (3.2%) | 0.001 |
|  |  | House | 8613 (96.4%) | 1810 (95.2%) |  |
|  |  | Missing | 159 (1.8%) | 31 (1.6%) |  |
|  |  |  |  |  |  |
|  | Home ownership | |  |  |  |
|  |  | Not owned by self | 742 (8.3%) | 201 (10.6%) | 0.005 |
|  |  | Owned by self | 8020 (89.8%) | 1669 (87.7%) |  |
|  |  | Missing | 173 (1.9%) | 32 (1.7%) |  |
|  |  |  |  |  |  |
|  | Lives alone | |  |  |  |
|  |  | No | 6304 (70.6%) | 1249 (65.7%) | <0.001 |
|  |  | Yes | 2552 (28.6%) | 637 (33.5%) |  |
|  |  | Missing | 79 (0.9%) | 16 (0.8%) |  |
|  |  |  |  |  |  |
|  | Own pet | |  |  |  |
|  |  | No | 5609 (62.8%) | 1186 (62.4%) | 0.94 |
|  |  | Yes | 3175 (35.5%) | 684 (36.0%) |  |
|  |  | Missing | 151 (1.7%) | 32 (1.7%) |  |
|  |  |  |  |  |  |
|  | In paid work | |  |  |  |
|  |  | No | 7897 (88.4%) | 1686 (88.6%) | 0.24 |
|  |  | Yes | 809 (9.1%) | 157 (8.3%) |  |
|  |  | Missing | 229 (2.6%) | 59 (3.1%) |  |
|  |  |  |  |  |  |
|  | Has care burden | |  |  |  |
|  |  | No | 7533 (84.3%) | 1544 (81.2%) | <0.001 |
|  |  | Yes | 1147 (12.8%) | 270 (14.2%) |  |
|  |  | Missing | 255 (2.9%) | 88 (4.6%) |  |
|  |  |  |  |  |  |
|  | Regular babysitting | |  |  |  |
|  |  | No | 7775 (87.0%) | 1666 (87.6%) | 0.003 |
|  |  | Yes | 959 (10.7%) | 173 (9.1%) |  |
|  |  | Missing | 201 (2.2%) | 63 (3.3%) |  |
|  |  |  |  |  |  |
|  | Support service usage | |  |  |  |
|  |  | No | 6980 (78.1%) | 1331 (70.0%) | <0.001 |
|  |  | Yes | 1930 (21.6%) | 569 (29.9%) |  |
|  |  | Missing | 25 (0.3%) | 2 (0.1%) |  |
|  |  |  |  |  |  |
|  | Has private health insurance | | |  |  |
|  |  | No | 2675 (29.9%) | 706 (37.1%) | <0.001 |
|  |  | Yes | 6260 (70.1%) | 1196 (62.9%) |  |
|  |  |  |  |  |  |
|  | Married | |  |  |  |
|  |  | No | 3029 (33.9%) | 725 (38.1%) | <0.001 |
|  |  | Yes | 5906 (66.1%) | 1177 (61.9%) |  |
|  |  |  |  |  |  |
|  | Completed high school | |  |  |  |
|  |  | No | 3155 (35.3%) | 727 (38.2%) | 0.03 |
|  |  | Yes | 5668 (63.4%) | 1147 (60.3%) |  |
|  |  | Missing | 112 (1.3%) | 28 (1.5%) |  |
|  |  |  |  |  |  |
|  | Community engagement | |  |  |  |
|  |  | Low | 1711 (19.1%) | 368 (19.3%) | 0.18 |
|  |  | High | 7044 (78.8%) | 1483 (78.0%) |  |
|  |  | Missing | 180 (2.0%) | 51 (2.7%) |  |
|  |  |  |  |  |  |
|  | Family/friends engagement | | |  |  |
|  |  | Low | 33 (0.4%) | 12 (0.6%) | 0.10 |
|  |  | High | 8876 (99.3%) | 1888 (99.3%) |  |
|  |  | Missing | 26 (0.3%) | 2 (0.1%) |  |
|  |  |  |  |  |  |
|  | Had major stress | |  |  |  |
|  |  | No | 4226 (47.3%) | 839 (44.1%) | 0.01 |
|  |  | Yes | 4709 (52.7%) | 1063 (55.9%) |  |
|  |  |  |  |  |  |

| **Supplementary Table 2:** Multivariable logistic regression analysis of emergency care usage for covariates selected by adaptive LASSO for all participants | | | | | | |
| --- | --- | --- | --- | --- | --- | --- |
|  |  |  |  |  |  |  |
|  |  |  |  | All participants n=10,379 | | |
|  |  |  |  | OR | 95%CI | p-value |
|  |  |  |  |  |  |  |
| Depressive symptoms indication | | | |  |  |  |
|  | CESD≤8 |  |  | Ref |  |  |
|  | CESD>8 |  |  | 1.35 | 1.11,1.64 | 0.003 |
|  |  |  |  |  |  |  |
| Biological factors | |  |  |  |  |  |
|  | Age group | |  |  |  |  |
|  |  | 70-72 |  | Ref |  |  |
|  |  | 73-75 |  | 1.11 | 0.98, 1.27 | 0.11 |
|  |  | 76-79 |  | 1.2 | 1.04, 1.39 | 0.01 |
|  |  | 80+ |  | 1.36 | 1.16, 1.60 | <0.001 |
|  |  |  |  |  |  |  |
|  | Gender |  |  |  |  |  |
|  |  | Male |  | Ref |  |  |
|  |  | Female |  | 0.84 | 0.75, 0.93 | 0.001 |
|  |  |  |  |  |  |  |
|  | PCS |  |  | 0.91 | 0.86, 0.97 | 0.002 |
|  |  |  |  |  |  |  |
|  | Polypharmacy >5 | |  |  |  |  |
|  |  | No |  | Ref |  |  |
|  |  | Yes |  | 1.33 | 1.18, 1.50 | <0.001 |
|  |  |  |  |  |  |  |
|  | Significant pain problems | | |  |  |  |
|  |  | No |  | Ref |  |  |
|  |  | Yes |  | 1.17 | 1.02, 1.35 | 0.028 |
|  |  |  |  |  |  |  |
|  | Falls in past year | |  |  |  |  |
|  |  | No |  | Ref |  |  |
|  |  | Yes |  | 1.18 | 1.06, 1.32 | 0.003 |
|  |  |  |  |  |  |  |
| Social factors | |  |  |  |  |  |
|  | SES |  |  |  |  |  |
|  |  | Lower 1-4 |  | Ref |  |  |
|  |  | Middle 5-7 |  | 0.88 | 0.77, 1.01 | 0.066 |
|  |  | Upper 8-10 |  | 0.97 | 0.84, 1.12 | 0.667 |
|  |  |  |  |  |  |  |
|  | Rurality |  |  |  |  |  |
|  |  | Not major city | | Ref |  |  |
|  |  | Major city |  | 0.84 | 0.74, 0.95 | 0.006 |
|  |  |  |  |  |  |  |
|  | House type | |  |  |  |  |
|  |  | Not house |  | Ref |  |  |
|  |  | House |  | 0.6 | 0.44, 0.82 | 0.001 |
|  |  |  |  |  |  |  |
|  | Living arrangement | |  |  |  |  |
|  |  | Not alone |  | Ref |  |  |
|  |  | Alone |  | 1.1 | 0.98, 1.24 | 0.109 |
|  |  |  |  |  |  |  |
|  | Own pet |  |  |  |  |  |
|  |  | No |  | Ref |  |  |
|  |  | Yes |  | 0.98 | 0.88, 1.09 | 0.745 |
|  |  |  |  |  |  |  |
|  | Support service usage | |  |  |  |  |
|  |  | No |  | Ref |  |  |
|  |  | Yes |  | 1.27 | 1.12, 1.44 | <0.001 |
|  |  |  |  |  |  |  |
|  | Has private health insurance | | |  |  |  |
|  |  | No |  |  |  |  |
|  |  | Yes |  | 0.79 | 0.71, 0.89 | <0.001 |
|  |  |  |  |  |  |  |
|  | Family/friends engagement | | |  |  |  |
|  |  | Low |  | Ref |  |  |
|  |  | High |  | 0.67 | 0.34, 1.33 | 0.254 |
|  |  |  |  |  |  |  |
|  |  |  |  |  |  |  |
|  |  |  |  |  |  |  |
